# Early prediction of ventilator-associated pneumonia in critical care patients: a machine learning model

**DOI:** 10.1186/s12890-022-02031-w

**Published:** 2022-06-25

**Authors:** Yingjian Liang, Chengrui Zhu, Cong Tian, Qizhong Lin, Zhiliang Li, Zhifei Li, Dongshu Ni, Xiaochun Ma

**Affiliations:** 1 Department of Critical Care Medicine, The First Hospital of China Medical University, North Nanjing Street 155, Shenyang, 110001 Liaoning Province China; 2 Philips Research China, 5F Building A2, 718 Ling Shi Road, Jing An District, Shanghai, 200072 China

**Keywords:** Ventilator-associated pneumonia, MIMIC database, Risk factors, Predictive modeling

## Abstract

**Background:**

This study was performed to develop and validate machine learning models for early detection of ventilator-associated pneumonia (VAP) 24 h before diagnosis, so that VAP patients can receive early intervention and reduce the occurrence of complications.

**Patients and methods:**

This study was based on the MIMIC-III dataset, which was a retrospective cohort. The random forest algorithm was applied to construct a base classifier, and the area under the receiver operating characteristic curve (AUC), sensitivity and specificity of the prediction model were evaluated. Furthermore, We also compare the performance of Clinical Pulmonary Infection Score (CPIS)-based model (threshold value ≥ 3) using the same training and test data sets.

**Results:**

In total, 38,515 ventilation sessions occurred in 61,532 ICU admissions. VAP occurred in 212 of these sessions. We incorporated 42 VAP risk factors at admission and routinely measured the vital characteristics and laboratory results. Five-fold cross-validation was performed to evaluate the model performance, and the model achieved an AUC of 84% in the validation, 74% sensitivity and 71% specificity 24 h after intubation. The AUC of our VAP machine learning model is nearly 25% higher than the CPIS model, and the sensitivity and specificity were also improved by almost 14% and 15%, respectively.

**Conclusions:**

We developed and internally validated an automated model for VAP prediction using the MIMIC-III cohort. The VAP prediction model achieved high performance based on its AUC, sensitivity and specificity, and its performance was superior to that of the CPIS model. External validation and prospective interventional or outcome studies using this prediction model are envisioned as future work.

**Supplementary Information:**

The online version contains supplementary material available at 10.1186/s12890-022-02031-w.

## Background

Ventilator-associated pneumonia (VAP) is the most common nosocomial pneumonia in critically ill patients [1]. The occurrence of VAP prolongs not only ventilator support but also stays in intensive care units (ICUs) and hospitals, thereby increasing healthcare costs and resulting in a poorer prognosis [2–4]. Studies have shown that some risk factors are associated with VAP. Some risk factors are patient-specific factors, such as age, pre-existing disease (chronic obstructive pulmonary disease, COPD) and a Glasgow coma score of 9 or less [5–7]. Other factors are care-related factors, such as head-of-the-bed angle, emergency intubation, aspiration, previous antibiotic treatment, and reintubation [5, 6, 8].

The early recognition of patients at a high risk of developing VAP and subsequent prevention of its progression are highly valuable in critical care units. Intensivists have been working on a VAP risk prediction model for several years. Several available prediction models are used to predict mortality in VAP patients [9–12]. The Clinical Pulmonary infection Score (CPIS, range from 0 to 12) is a score based on general parameters(body temperature, leukocyte count, volume and character of tracheal secretions, arterial oxygenation, chest X-ray, and culture of tracheal aspirate), it has moderate to good accuracy in VAP prediction and is simple and easy to perform and often used in clinical diagnosis of VAP [13, 14]. However, there is no early risk prediction model for VAP.

Machine learning algorithms have become more important tools since they can be more accurate than traditional logistic regression, which has been suggested by previous comparison studies [15, 16]. Of all machine learning algorithms, the random forest for regression and classification has considerably gained popularity. It is an “ensemble learning” technique consisting of the aggregation of a large number of decision trees. For classification tasks, the output of the random forest is the class selected by most trees and for regression tasks, the mean or average prediction of the individual trees is returned, resulting in a better performance and reduction of variance [16]. The study applied the random forest algorithm to construct a base classifier for early prediction of ventilator-associated pneumonia in critical care patients.

The aim of this study was to use the Medical Information Mart for Intensive Care (MIMIC)-III dataset to develop and validate machine learning models for the early discrimination of patients at a high risk of VAP 24 h after intubation and assess its prognostic accuracy. The MIMIC database is an open, large, single-center database that can be used freely by researchers worldwide, and it has been widely used in the development of predictive models, epidemiological studies, and educational courses [17]. Also, We also compare the performance of Clinical Pulmonary Infection Score (CPIS)-based model (threshold value ≥ 3) using the same training and test data sets.

## Methods

### Datasets

The MIMIC-III database was used to train, validate and test the models and comprises unidentified health-related data associated with 61,532 ICU stays in multiple critical care units in Beth Israel Deaconess Medical Center between 2001 and 2012 [17]. This database is a publicly available database constructed in compliance with the Health Insurance Portability and Accountability Act. The study protocol was approved by the ethics committee of the First Hospital of China Medical University (No. 2019–197-2).

### Data annotation and extraction

**Fig. 1:**
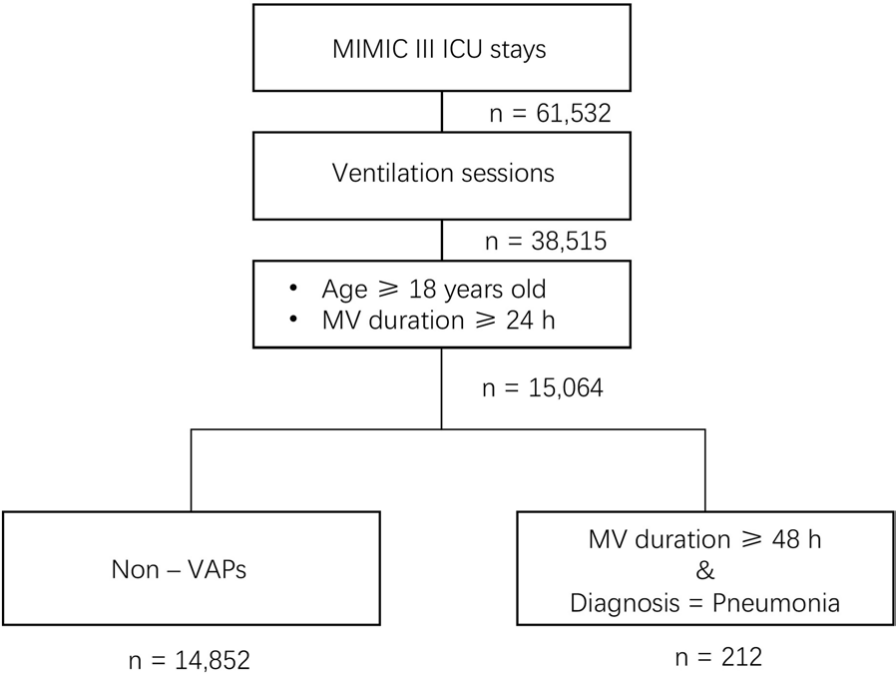
Study profile. MIMIC, Medical Information Mart for Intensive Care; MV Mechanical ventilation; VAP, ventilator-associated pneumonia; ICU, intensive care unit

In total, 38,515 ventilation sessions were identified in the MIMIC-III database and filtered according to the patient inclusion process depicted in Fig. 1. In total, 10,431 patients aged over 18 years who received mechanical ventilation for longer than 24 h were included in this study. Pneumonia occurring > 48 h after endotracheal intubation and mechanical ventilation were annotated as VAP according to the VAP definition [18]. The other sessions were grouped as non-VAP sessions. When VAP was diagnosed, the presence of infection at other sites was recorded.

**Fig. 2:**
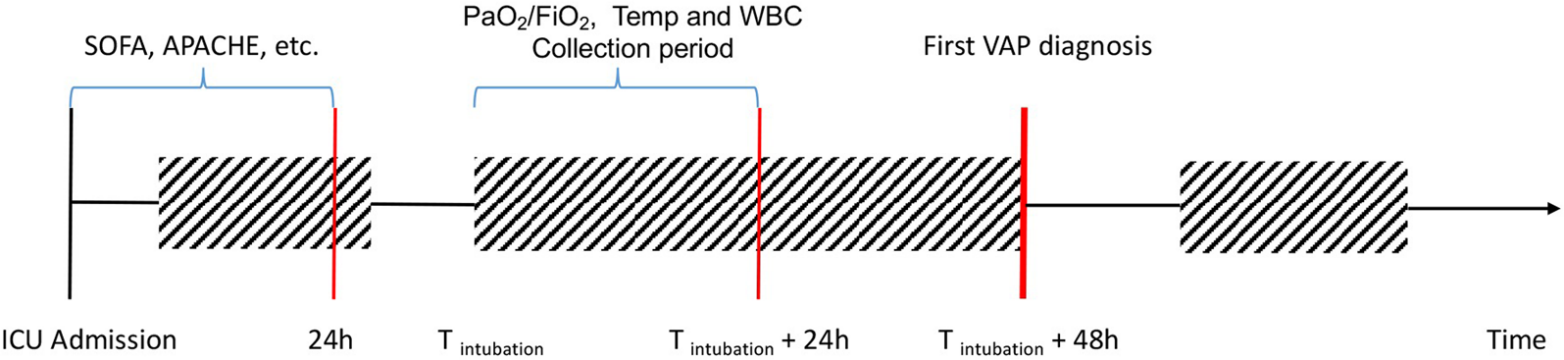
Timeline for the first VAP prediction and VAP variable extraction. ICU, intensive care unit; SOFA, Sequential Organ Failure Assessment; APACHE, Acute Physiology and Chronic Health Evaluation; PaO_2_/FiO_2_, the partial pressure of arterial oxygen/ fraction of inspired oxygen; WBC, white blood cell count; VAP, ventilator-associated pneumonia

To detect the risk of the first occurrence of VAP early, a set of 42 variables (features) were extracted from the MIMIC-III dataset according to our previous studies and literature [5–8, 19], including age, sex, admission source [medical intensive care unit (MICU), others ( CCU (Coronary Care Unit), SICU(Surgical Intensive Care Unit), CSRU(Cardiac Surgery Recovery Unit) and ISICU(Trauma Surgical Intensive Care Unit))] and type (emergency, elective), reintubation, pre-existing diseases, the worst value of the partial pressure of the arterial oxygen/fraction of inspired oxygen (PaO_2_/FiO_2_) ratio, white blood cell count (WBC), body temperature in the first 24 h after ventilation, the worst value of the APACHE III and its subcomponents, the sequential organ failure assessment (SOFA) and its subcomponents in the first 24 h after admission to the ICU, coma, aspiration, sepsis, bacteremia, trauma/polytrauma, fracture and pneumothorax (the detailed information of these 42 variables is provided in Additional file 4: Table S1). Figure 2 shows the timeline for VAP diagnosis and VAP variable extraction.

### Data splitting and sampling

**Fig. 3:**
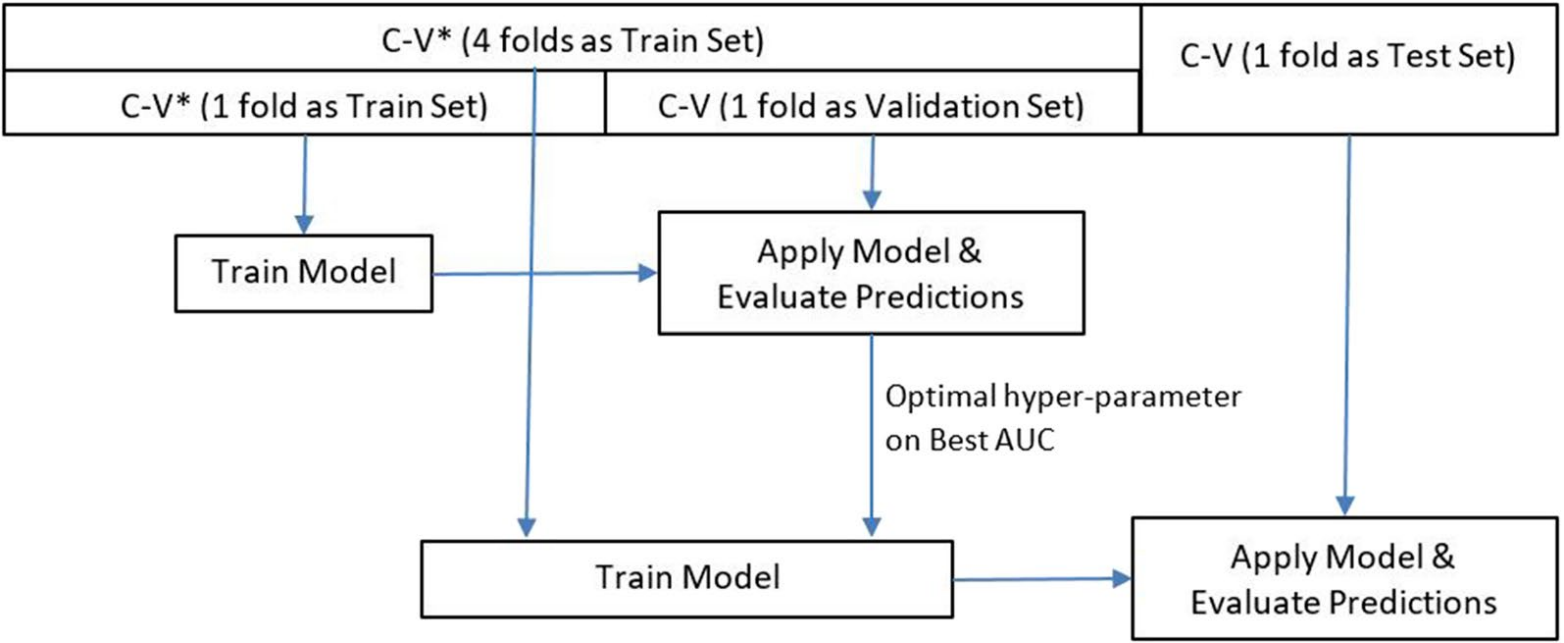
Model training, validation and testing pipeline. The dataset was divided into four groups as a training dataset and one group as test dataset for five-fold cross-validation. C-V, cross-validation

Figure 3 describes the pipeline applied for the model training, validation and testing. The included dataset was divided into a training dataset and test dataset for the five-fold cross-validation in which four folds were used as the training dataset, the remaining fold was used as the test dataset, and the folds were mutually exclusive. To identify the optimal hyperparameter of the model, two-fold cross-validation was performed using the training dataset, and then, the model was retrained using the optimal hyperparameter based on the entire training dataset to learn the model parameters. Due to an extreme imbalance between the number of non-VAP and VAP patients, the negative dataset was divided into 100 subgroups for resampling. Stratified sampling was used to ensure an even class distribution.

### Data preprocessing

Additional file 1: Fig. S1 shows the data preprocessing steps. For the numeric variables, if a patient did not have a measurement, the missing value was filled by using the median interpolation of the whole cohort (Additional file 4: Table S1 shows the count and percentage of missing data in the VAP group and non-VAP group; Fisher’s exact test was used to test the significance). For the categorical variables with *d* categories, the raw data were mapped to a d-dimensional vector, where each dimension corresponded to a different category; however, the categorical variables with two categories (e.g., sex = {F, M}) were sufficiently mapped to {0, 1}. Then, both the numeric and categorical data were normalized for the training dataset, which required min–max feature scaling to adjust for variable values measured on different scales.

### Model development and performance measurement

Since there were many more non-VAP instances than VAP instances, we divided the non-VAP instances into 100 subgroups with mutual exclusivity. One subgroup of non-VAP instances was combined with the VAP dataset to train one model; then, 100 models were combined based on the performance average or major voting as the final model. The ensemble method was applied to 100 subgroups of non-VAP instances in combination with VAP instances as shown in Additional file 2: Fig. S2.

The random forest algorithm was applied to construct the classifier. The area under the receiver operating characteristic (ROC) curve (AUC), accuracy, sensitivity and specificity of the prediction model were evaluated. Furthermore, we used an original CPIS-based model for the early detection of VAP as a benchmark model to compare with our machine learning model, and the performance of the classification model was evaluated using the same training and test datasets. The performance is described as the mean ± SD to indicate the performance distribution of the subgroups, and the SD was used to determine whether any overfitting of the model occurred in certain datasets.

The Bayes search method was applied to fine-tune the hyperparameters of the base classifier using the validation set. In the random forest classifier, the optimal number of estimators of the hyperparameter was adjusted to 104, which was randomly obtained via Bayes search in the range from 1 to 300.

### Statistical analysis

In the analysis of the clinical characteristics of both the VAP and non-VAP groups, the numeric variables are described as medians and interquartile ranges (IQRs; represented by the 25th and 75th percentile values), and the categorical variables are described as counts and percentages. To compare the two groups, we used Fisher’s exact test for the categorical variables and the Mann–Whitney U-test for the numeric variables. A *p*-value less than 0.05 was considered statistically significant. Python3.0 was used to perform the statistical, sklearn.model was used to perform model building.

## Results

According to the screening criteria shown in Fig. 1, 38,515 ventilation sessions were included with 212 VAP sessions between 2001 and 2012 in the MIMIC-III cohort, and the incidence density was 2 per 1,000 ventilator-days. The median time on mechanical ventilation from endotracheal intubation to the first VAP episode was 5.4 days (IQR, 3.2 days to 8.5 days). None of these VAP patients had infections in other sites. The missing counts and percentages of the 42 variables in the overall, VAP, and non-VAP groups are shown in Additional file 4: Table S1. Compared with the overall study cohort, the non-VAP group had significantly higher missing albumin and acid–base scores in the APACHE III and respiration scores in SOFA. However, the VAP group had a higher missing percentage of pulmonary alveolus-arterial difference of oxygen pressure/partial pressure of oxygen (A-aDO_2_/PaO_2_) and urine output.

The univariate analysis indicated that compared to the control group, the VAP group in the study cohort had a significantly different admission source and type (*p* < 0.001); specifically, the VAP group had a significantly higher ratio of patients from the MICU, and only one VAP patient was not transferred from the emergency department (see details in Table 1). The worst value of the PaO_2_/FiO_2_ ratio in the first 24 h after ventilation was significantly deteriorated (*p* < 0.001) in the VAP group compared with that in the control group. The reintubation ratio did not significantly differ (*p* = 0.823) between the VAP group and non-VAP group, whereas the VAP group demonstrated a significantly higher ratio in aspiration (*p* = 0.004). Regarding pre-existing diseases, there was no difference between the VAP group and non-VAP group, except for hypertension.

**Table 1:** Demographic and clinical characteristics of study cohort in MIMIC III

	Overall (n = 10,431)	VAP group (n = 212)	Non-VAP group (n = 10,219)	*p* value
Age(years), median(IQR)	66.3 (53.1–76.0)	66.3 (52.1–77.8)	66.3 (53.1–76.0)	0.387
Gender, n(%)				0.107
Male	5937 (56.9)	109 (51.4)	5828 (57.0)	
Female	4494 (43.1)	103 (48.6)	4391 (43.0)	
Admission source, n(%)				< 0.001
MICU	4089 (39.2)	154 (72.6)	3935 (38.5)	
Other ICU	6342 (60.8)	58 (27.4)	6284 (61.5)	
Admission type, n(%)				< 0.001
Emergency	9504 (91.1)	211 (99.5)	9293 (90.9)	
Elective	927 (8.9)	1 (0.5)	926 (9.1)	
Reintubation, n(%)	3294 (31.6%)	65 (30.7%)	3229 (30.6%)	0.823
Pre-existing Diseases, n(%)				
COPD	101 (1.0%)	2 (0.9%)	99 (1.0)	1.0
Diabetes	2698 (25.9)	60 (28.3)	2638 (25.8)	0.428
Hypertension	4943 (47.4)	80 (37.7)	4863 (47.6)	0.004
Solid tumor	313 (3.0)	8 (3.8)	305 (3.0)	0.537
Metastatic tumor	418 (4.0)	5 (2.4)	413 (4.0)	0.286
Renal failure	1721 (16.5)	26 (12.3)	1695 (16.6)	0.111
Liver failure	1653 (15.9)	22 (10.4)	1631 (16.0)	0.028
PaO_2_/FiO_2_, median(IQR)	240.0 (178.5–315.4)	194.17 (150.0–256.5)	241.0 (179.4–316.7)	< 0.001
WBC(K/uL), median(IQR)	12.8 (9.2–17.7)	13.2 (9.8–18.0)	12.8 (9.2–17.7)	0.121
Body temperature(℃), median(IQR)	37.8 (37.3–38.4)	37.9 (37.3–38.6)	37.8 (37.3–38.4)	0.255
APACHE III score, median(IQR)	50.0 (37.0–66.0)	53.0 (40.75–64.0)	50.0 (37.0–66.0)	0.031
HR	5.0 (0.0–7.0)	5.0 (0.0–7.0)	5.0 (0.0–7.0)	0.028
MAP	15.0 (7.0–15.0)	15.0 (7.0–15.0)	15.0 (7.0–15.0)	0.197
Temperature	0.0 (0.0–2.0)	0.0 (0.0–2.0)	0.0 (0.0–2.0)	< 0.001
RR	6.0 (0.0–6.0)	6.0 (0.0–9.0)	6.0 (0.0–6.0)	0.001
A-aDO_2_/PaO_2_	0.0 (0.0–0.0)	0.0 (0.0–0.0)	0.0 (0.0–0.0)	< 0.001
Hematocrit	3.0 (3.0–3.0)	3.0 (3.0–3.0)	3.0 (3.0–3.0)	0.269
WBC	0.0 (0.0–1.0)	0.0 (0.0–1.0)	0.0 (0.0–1.0)	0.083
Creatinine	0.0 (0.0–7.0)	0.0 (0.0–7.0)	0.0 (0.0–7.0)	0.484
UO	5.0 (0.0–7.0)	5.0 (0.0–7.0)	5.0 (0.0–7.0)	0.013
BUN	7.0 (2.0–11.0)	7.0 (2.0–11.0)	7.0 (2.0–11.0)	0.094
Sodium	0.0 (0.0–2.0)	0.0 (0.0–2.0)	0.0 (0.0–2.0)	0.016
ALB	0.0 (0.0–6.0)	0.0 (0.0–6.0)	0.0 (0.0–6.0)	0.087
Bilirubin	0.0 (0.0–0.0)	0.0 (0.0–0.0)	0.0 (0.0–0.0)	0.008
Glucose	0.0 (0.0–3.0)	3.0 (0.0–3.0)	0.0 (0.0–3.0)	0.017
Acid–base	3.0 (1.0–6.0)	3.0 (1.0–5.0)	3.0 (1.0–6.0)	0.077
GCS	0.0 (0.0–0.0)	0.0 (0.0–0.0)	0.0 (0.0–0.0)	0.017
SOFA score, median(IQR)	6.0 (4.0–9.0)	6.0 (4.0–8.0)	6.0 (4.0–9.0)	0.034
Respiration	3.0 (0.0–3.0)	3.0 (2.0–3.0)	3.0 (0.0–3.0)	0.014
Coagulation	0.0 (0.0–1.0)	0.0 (0.0–1.0)	0.0 (0.0–1.0)	< 0.001
Liver	0.0 (0.0–1.0)	0.0 (0.0–0.0)	0.0 (0.0–1.0)	0.004
Cardiovascular	1.0 (1.0–3.0)	1.0 (1.0–3.0)	1.0 (1.0–3.0)	0.022
CNS	0.0 (0.0–1.0)	0.0 (0.0–0.0)	0.0 (0.0–1.0)	0.008
Renal	1.0 (0.0–2.0)	1.0 (0.0–2.0)	1.0 (0.0–2.0)	0.471
Coma adm, n(%)	6 (0.1%)	0 (0.0%)	6 (0.1%)	1.0
Aspiration adm, n(%)	32 (0.3%)	4 (1.9%)	28 (0.3%)	0.004
Sepsis adm, n(%)	548 (5.3%)	10 (4.7%)	538 (5.3%)	0.876
Bacteremia adm, n(%)	11 (0.1%)	0 (0.0%)	11 (0.1%)	1.0
Trauma adm, n(%)	202 (1.9%)	0 (0.0%)	202 (2.0%)	0.037
Polytrauma adm, n(%)	45 (0.4%)	0 (0.0%)	45 (0.4%)	1.0
Fracture adm, n(%)	27 (0.3%)	0 (0.0%)	27 (0.3%)	1.0
Pneumothorax adm, n(%)	19 (0.2%)	0 (0.0%)	19 (0.2%)	1.0

MICU medical intensive care unit, APACHE III Acute Physiology and Chronic Health Evaluation III, PaO_2_/FiO_2_ the partial pressure of arterial oxygen/ fraction of inspired oxygen, WBC white blood cell count, HR heart rate, MAP mean arterial pressure, RR respiratory rate, A-aDO_2_/PaO_2_ pulmonary alveolus-arterial difference of oxygen pressure/ partial pressure of oxygen, UO urine output, BUN blood urea nitrogen, ALB albumin, GCS Glasgow Coma Scale, SOFA sequential organ failure assessment, CNS central nervous system, COPD chronic obstructive pulmonary disease, adm admission

**Fig. 4:**
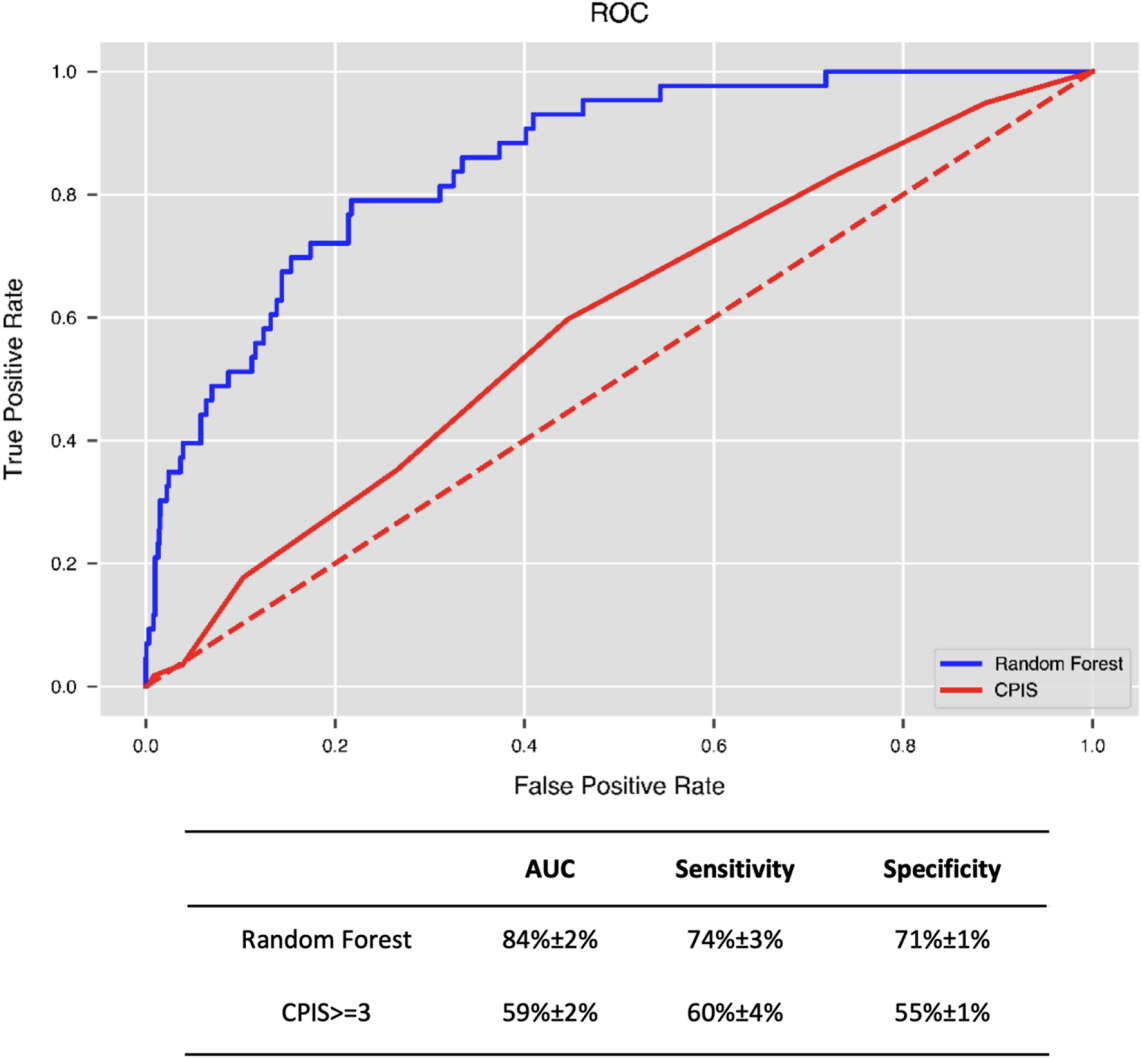
Performances of the VAP predictive model and CPIS model

**Fig. 5:**
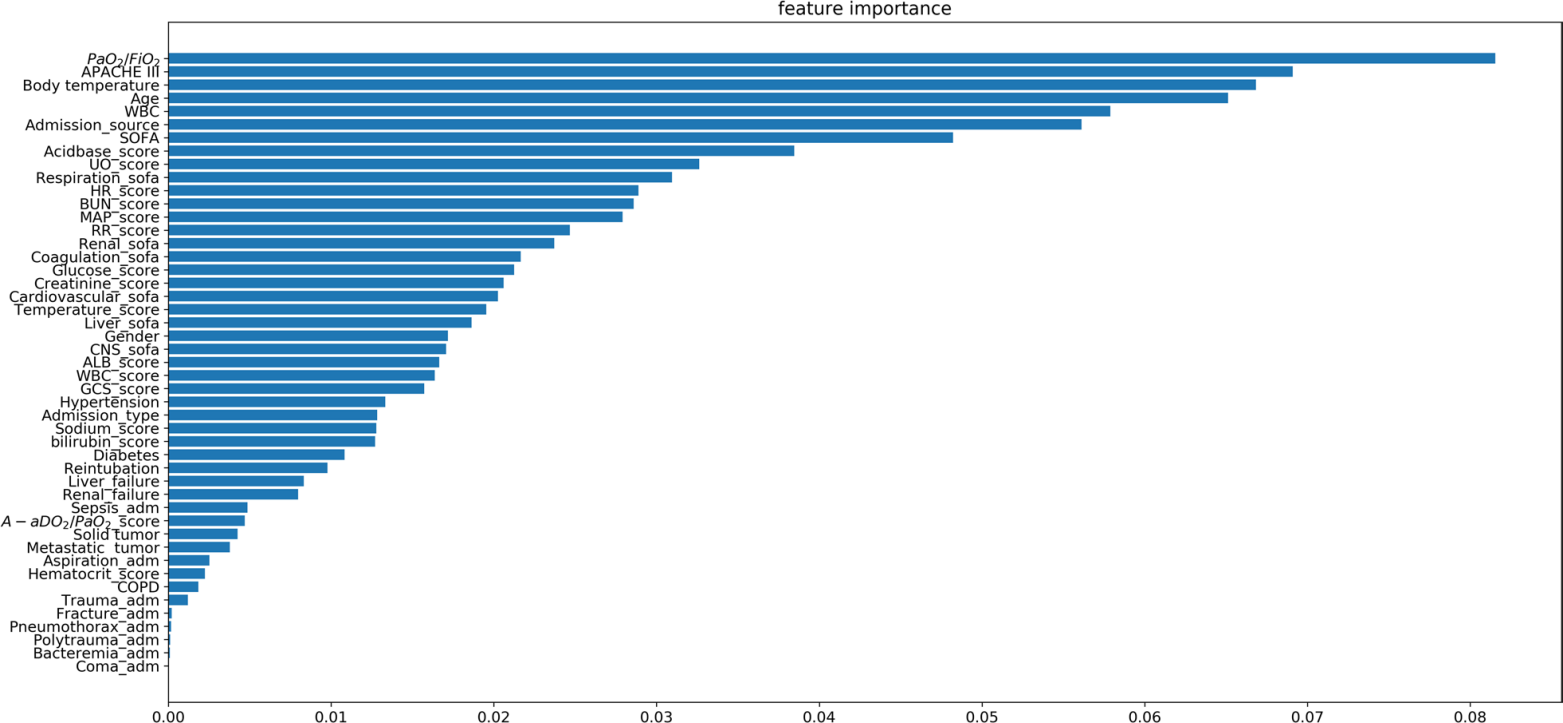
Feature importance in our predictive model of VAP. The feature importance of the optimal random forest model indicates the features’ contribution to the VAP prediction

Figure 4 shows that the AUC of the optimal performance corresponding to the random forest model was 84% ± 2% in the validation using the pure testing datasets, and the sensitivity and specificity approached 74 ± 3% and 71 ± 1%, respectively. Using the same test datasets, the best performance of the CPIS-based model was AUC = 59 ± 2%, sensitivity = 60± 4%, and specificity = 55± 1% as CPIS equal to or greater than 3. Figure 5 shows the feature importance of the optimal random forest model, indicating the contribution rankings of the features to the prediction value in the model. The admission source, APACHE III and SOFA scores along with their sub items, age, worst body temperature value, PaO_2_/FiO_2_ ratio, and WBC in the initial 24 h after ventilation were the top 10 most important features and contributed over 46% of the total prediction value. The respiration items of the SOFA were the highest contributors to the total SOFA score (4% of the VAP prediction model), indicating the significance of respiration for organ failure.

## Discussion

In this retrospective cohort study, we developed and validated a machine learning model for the early detection of VAP patients in the first 24 h after intubation. The final predictive AUC showed a good performance (AUC: 84%, sensitivity: 74%, and specificity: 71%) as an AUC value between 75 and 92% indicates good diagnostic capability [20]. Additionally, our VAP machine learning model achieved better results than the CPIS-based model by almost 25%, and the sensitivity and specificity were improved by almost 14% and 15%, respectively.

A CPIS score threshold of 6 helps to distinguish the presence or absence of pulmonary infection [21]. But in our MIMIC III cohort data, a CPIS score of 6 did not show a good performance. Considering the heterogeneity in the performances of CPIS for the diagnosis of VAP in ventilated patients [13, 14], different score thresholds were tested to determine the best performance. Additional file 3: Fig. S3 shows that when the score was equal to or greater than 3, the CPIS-based model had the best performance. It is for this reason that we have compared our model with a CPIS score ≥ 3 instead of with a CPIS score ≥ 6.

Low PaO_2_/FiO_2_ ratio is one of the main clinical manifestations of ARDS. The typical ARDS manifestations include increased pulmonary vascular permeability, pulmonary edema and alveolar trapping, which lead to refractory hypoxia and decreased pulmonary compliance [22]. The relationship between ARDS and the subsequent development of VAP is complex. In mechanically-ventilated patients, the cyclic stretching of lung cells induces acidification of the milieu, which promotes bacterial growth [23]. Injurious mechanical ventilation may promote the lungs to release cytokines [24, 25]. In addition, alveolar macrophages and neutrophils exhibit reduced bacterial phagocytosis and killing, thereby affecting the lung and systemic antibacterial defenses [24, 26, 27].

We found that the APACHE III and SOFA scores greatly contributed to the final predictive model. The APACHE scoring system is used to describe the severity of illness and predict the outcome of critically ill patients. The APACHE II and III are widely employed in the ICU [28, 29], and the overall goodness-of-fit of the two predictive models was similar. APACHE III expanded the acute physiology score project compared to APACHE II, added the following six parameters: blood urea nitrogen, total bilirubin, blood glucose, albumin, artery CO_2_ partial pressure (PaCO_2_) and urine output. These six parameters are more responsive in clinical practice [30, 31]. The APACHE II was better in predicting risk among surgical patients and patients with gastrointestinal disease [30], while the APACHE III score was a good predictor of internal medical conditions and nosocomial pneumonia [31, 32].

Reintubation, aspiration, COPD, trauma, and coma are usually the risk factors of VAP. In our model, only first VAP sessions were accounted for the prediction to avoid intra-correlation between consecutive sessions. That is why reintubation were not in the higher ranking. For aspiration, COPD, trauma and coma, we only included diagnosis in admission as predictor, and then, the ratio in both VAP and non-VAP group is quite low (< 2%, details in Table 1).

A major limitation of this study was that a small number of VAP cases were delayed or missed for various reasons, resulting in a false negative diagnosis of VAP. We acquired the infections sites by using nursing chart. It was possible to be underrecognized or not charted by nurses. Sputum examination is necessary when VAP is suspected. Sputum frequency is reported to be a factor in the VAP prediction model. The definition of VAP has greatly evolved over the last two decades and different definitions are used in clinical practice. Our model is developed based on definitions used between 2001 and 2012. Our solution to circumvent this problem is to take the current definitions of VAP(18) and use a data driven approach to label patients as VAP or non-VAP. This would solve the problem of outdated definitions, time stamping and subjectivity. In our study, the non-VAP group included patients with mechanical ventilation for 24 h rather than patients with 48 h of mechanical ventilation for the following reasons: we selected the worst body temperature values, PaO_2_/FiO_2_ ratio, and WBC during the initial 24 h after ventilation and the worst values of the APACHE III and SOFA scores in the first 24 h after admission to the ICU as VAP predictors. If we included patients with 48 h of mechanical ventilation in the control group, some non-VAP patients could be missed. Our predictive model can provide risk stratification for VAP patients within independently-defined patient groups. Prevention guidelines have been developed to allow higher-risk patients to benefit from more aggressive strategies or adjuvant therapy. Additionally, a longer prediction lead time could increase the likelihood that a patient can benefit from early intervention.

## Conclusions

We developed and internally validated an automated model for VAP prediction using the MIMIC-III cohort. The VAP prediction model achieved a high performance based on the AUC, sensitivity and specificity, and its performance was superior to that of the CPIS-based model. External validation and prospective interventional or outcome studies using this prediction model are envisioned as future work.

## Supplementary Information

**Additional file 1:** Data pre-processing pipeline.

**Additional file 2:** Imbalanced dataset model. The non-VAP dataset was divided into 100 subgroups, one of which was combined with the VAP dataset to train the model, and then, 100 models were combined into the final model.

**Additional file 3:** Performance of CPIS-based model in MIMICIII cohort and selection of optimal threshold selection. From CPIS was a score (t) ranged from 0-12 with 6 subcategories. Performance of CPIS was indicated by using AUROC along with the increase of t. Considering t could only be integer, i.e. t=2,3,4,…, t=3 was selected as cut-off since the drop of sensitivity and increase of specificity could be balanced, i.e. the point with largest Youden index.

**Additional file 4:** List of 42 variables and missing value in study cohort.

## Data Availability

The datasets generated and/or analysed during the current study are available in the MIMIC III repository. [https://mimic.mit.edu/].

## Abbreviations

VAP: Ventilator-associated pneumonia
ICU: Intensive care unit
APACHE III: Acute physiology and chronic health evaluation III
MIMIC: Medical Information Mart for Intensive Care
SOFA: Sequential organ failure assessment
PaO_2_/FiO_2_: The partial pressure of arterial oxygen/fraction of inspired oxygen
WBC: White blood cell count
COPD: Chronic obstructive pulmonary disease
ARDS: Acute respiratory distress syndrome
CPIS: Clinical pulmonary infection score
