# Supplementary figures and images for: Early prediction of ventilator-associated pneumonia in critical care patients: a machine learning model
Source: BMC Pulm Med. 2022 Jun 25;22:250. doi: 10.1186/s12890-022-02031-w (PMC9233772; doi:10.1186/s12890-022-02031-w)

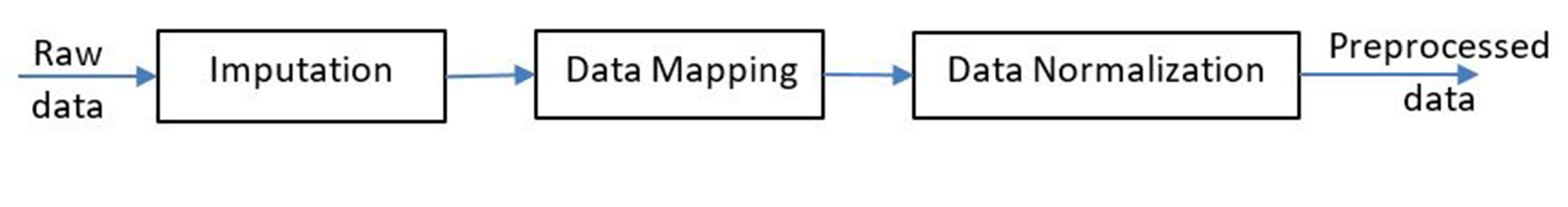

Supplement: Supplementary file 1 — Additional file 1: Data pre-processing pipeline. [file 12890_2022_2031_MOESM1_ESM.tif]

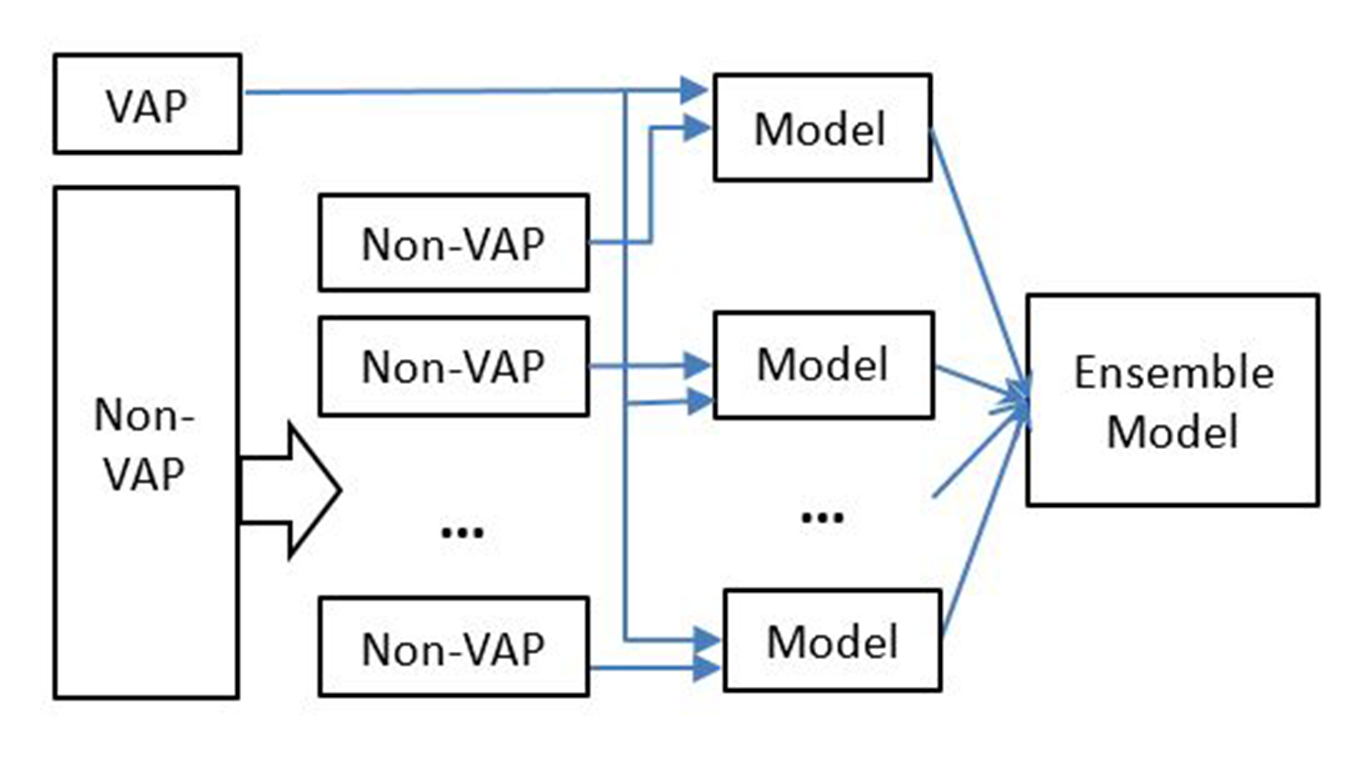

Supplement: Supplementary file 2 — Additional file 2: Imbalanced dataset model. The non-VAP dataset was divided into 100 subgroups, one of which was combined with the VAP dataset to train the model, and then, 100 models were combined into the final model. [file 12890_2022_2031_MOESM2_ESM.tif]

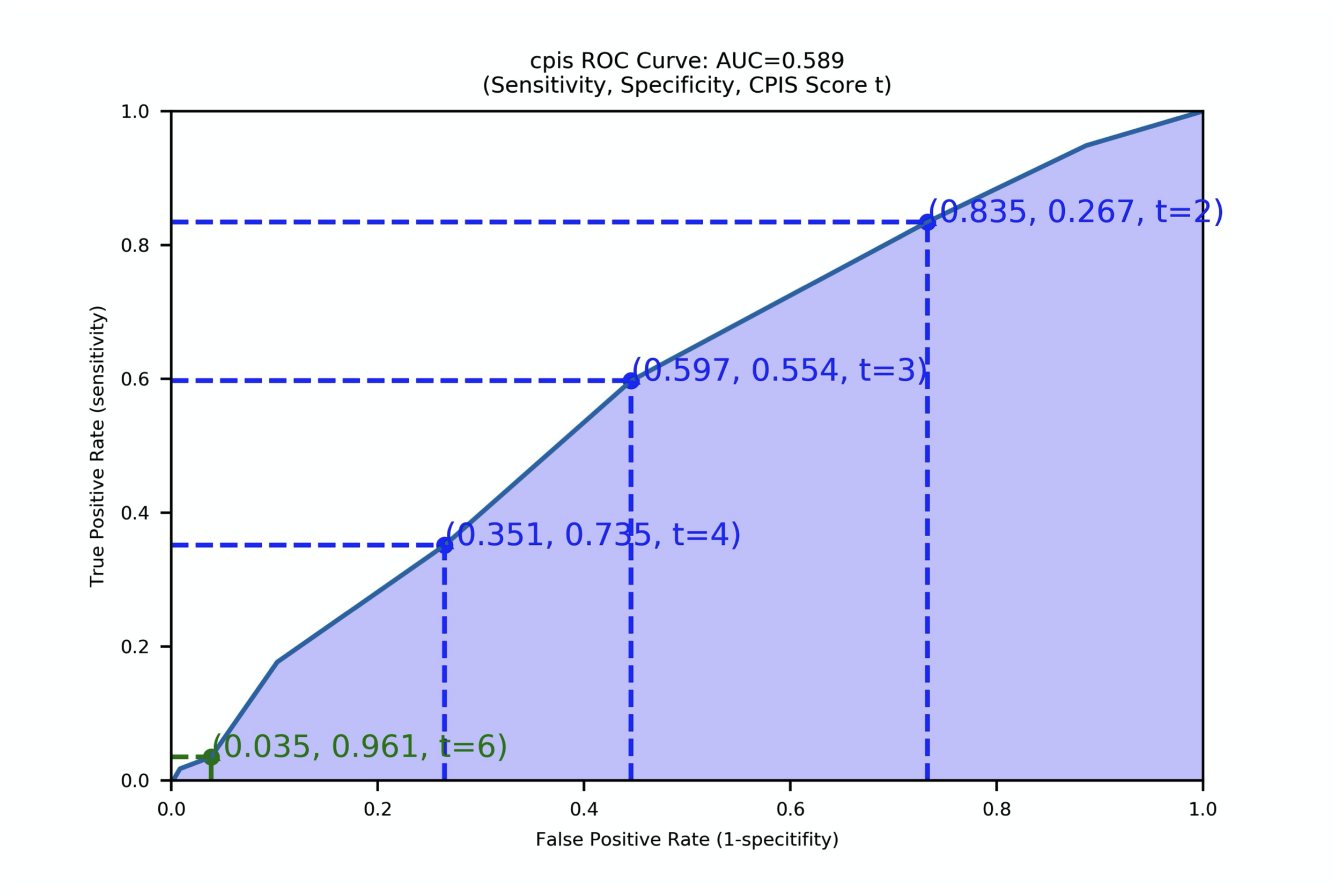

Supplement: Supplementary file 3 — Additional file 3: Performance of CPIS-based model in MIMICIII cohort and selection of optimal threshold selection. From CPIS was a score (t) ranged from 0-12 with 6 subcategories. Performance of CPIS was indicated by using AUROC along with the increase of t. Considering t could only be integer, i.e. t=2,3,4,…, t=3 was selected as cut-off since the drop of sensitivity and increase of specificity could be balanced, i.e. the point with largest Youden index. [file 12890_2022_2031_MOESM3_ESM.tiff]
