# Supplementary material for: Early prediction of ventilator-associated pneumonia in critical care patients: a machine learning model
Source: BMC Pulm Med. 2022 Jun 25;22:250. doi: 10.1186/s12890-022-02031-w (PMC9233772; doi:10.1186/s12890-022-02031-w)
Supplement: Supplementary file 4 — Additional file 4: List of 42 variables and missing value in study cohort. [file 12890_2022_2031_MOESM4_ESM.docx]

**Table S1**, List of 42 variables and missing value in study cohort

|  | **Overall**  **(n = 10431)** | **VAP group**  **(n = 212)** | **Non-VAP group**  **(n = 10219)** | ***p* value** |
| --- | --- | --- | --- | --- |
| Age | 0 (0.0%) | 0 (0.0%) | 0 (0.0%) | 1.0 |
| Gender | 0 (0.0%) | 0 (0.0%) | 0 (0.0%) | 1.0 |
| Admission source | 0 (0.0%) | 0 (0.0%) | 0 (0.0%) | 1.0 |
| Admission type | 0 (0.0%) | 0 (0.0%) | 0 (0.0%) | 1.0 |
| Reintubation | 0 (0.0%) | 0 (0.0%) | 0 (0.0%) | 1.0 |
| Pre-existing Diseases  COPD | 0 (0.0%) | 0 (0.0%) | 0 (0.0%) | 1.0 |
| Diabetes | 0 (0.0%) | 0 (0.0%) | 0 (0.0%) | 1.0 |
| Hypertension | 0 (0.0%) | 0 (0.0%) | 0 (0.0%) | 1.0 |
| Renal failure | 0 (0.0%) | 0 (0.0%) | 0 (0.0%) | 1.0 |
| Liver failure | 0 (0.0%) | 0 (0.0%) | 0 (0.0%) | 1.0 |
| Solid tumor | 0 (0.0%) | 0 (0.0%) | 0 (0.0%) | 1.0 |
| Metastatic tumor | 0 (0.0%) | 0 (0.0%) | 0 (0.0%) | 1.0 |
| PaO_2_/FiO_2_ | 876 (8.4%) | 17 (8.0%) | 859 (8.4%) | 1.0 |
| WBC | 128 (1.2%) | 3 (1.4%) | 125 (1.2%) | 0.746 |
| Body temperature | 213 (2.0%) | 0 (0.0%) | 213 (2.1%) | 0.024 |
| APACHE III | 0 (0.0%) | 0 (0.0%) | 0 (0.0%) | 1.0 |
| HR score | 47 (0.5%) | 2 (0.9%) | 45 (0.4%) | 0.248 |
| MAP score | 49 (0.5%) | 2 (0.9%) | 47 (0.5%) | 0.263 |
| Temperature score | 257 (2.5%) | 2 (0.9%) | 255 (2.5%) | 0.181 |
| RR score | 47 (0.5%) | 2 (0.9%) | 45 (0.4%) | 0.248 |
| A-aDO_2_/PaO_2_ score | 7124 (68.3%) | 170 (80.2%) | 6954(68.1%) | <0.001 |
| Hematocrit score | 27 (0.3%) | 0 (0.0%) | 27 (0.3%) | 1.0 |
| WBC score | 54 (0.5%) | 0 (0.0%) | 54 (0.5%) | 0.629 |
| Creatinine score | 19 (0.2%) | 0 (0.0%) | 19 (0.2%) | 1.0 |
| UO score | 573(5.5%) | 20 (9.4%) | 553(5.4%) | 0.021 |
| BUN score | 19 (0.2%) | 0 (0.0%) | 19 (0.2%) | 1.0 |
| Sodium score | 19(0.2%) | 0 (0.0%) | 19(0.2%) | 1.0 |
| ALB score | 5069(48.6%) | 80 (37.7%) | 4989 (48.8%) | 0.001 |
| Bilirubin score | 4153 (39.8%) | 74 (34.9%) | 4079 (53.9%) | 0.156 |
| Glucose score | 9 (0.1%) | 0 (0.0%) | 9(0.1%) | 1.0 |
| Acid-base score | 735 (7.1%) | 6 (2.8%) | 729 (7.1%) | 0.014 |
| GCS score | 210 (2.0%) | 5 (2.4%) | 205 (2.0%) | 0.62 |
| SOFA | 0 (0.0%) | 0 (0.0%) | 0 (0.0%) | 1.0 |
| Respiration sofa | 1500 (14.4%) | 15 (7.1%) | 1485 (14.5%) | 0.001 |
| Coagulation sofa | 38 (0.4%) | 0 (0.0%) | 38 (0.4%) | 1.0 |
| Liver sofa | 4153 (39.8%) | 74 (34.9%) | 4079 (39.9%) | 0.156 |
| Cardiovascular sofa | 49 (0.5%) | 2 (0.9%) | 47 (0.5%) | 0.263 |
| CNS sofa | 54 (0.5%) | 3 (1.4%) | 51 (0.5%) | 0.097 |
| Renal sofa | 7(0.1%) | 0 (0.0%) | 7(0.1%) | 1.0 |
| Coma adm | 0 (0.0%) | 0 (0.0%) | 0 (0.0%) | 1.0 |
| Aspiration adm | 0 (0.0%) | 0 (0.0%) | 0 (0.0%) | 1.0 |
| Sepsis adm | 0 (0.0%) | 0 (0.0%) | 0 (0.0%) | 1.0 |
| Bacteremia adm | 0 (0.0%) | 0 (0.0%) | 0 (0.0%) | 1.0 |
| Trauma adm | 0 (0.0%) | 0 (0.0%) | 0 (0.0%) | 1.0 |
| Polytrauma adm | 0 (0.0%) | 0 (0.0%) | 0 (0.0%) | 1.0 |
| Fracture adm | 0 (0.0%) | 0 (0.0%) | 0 (0.0%) | 1.0 |
| Pneumothorax adm | 0 (0.0%) | 0 (0.0%) | 0 (0.0%) | 1.0 |

*Abbreviations*: PaO_2_/FiO_2_ the partial pressure of arterial oxygen/ fraction of inspired oxygen, WBC white blood cell count, APACHE III Acute Physiology and Chronic Health Evaluation III, HR heart reat, MAP mean arterial pressure, RR respiratory rate, A-aDO_2_/PaO_2_ pulmonary alveolus-arterial difference of oxygen pressure/ partial pressure of oxygen, UO urine output, BUN blood urea nitrogen, ALB albumin, GCS Glasgow Coma Scale, SOFA sequential organ failure assessment, CNS central nervous system, COPD chronic obstructive pulmonary disease, adm admission
